# 4-Hydroxyestradiol induces mammary epithelial cell transformation through Nrf2-mediated heme oxygenase-1 overexpression

**DOI:** 10.18632/oncotarget.10516

**Published:** 2016-07-09

**Authors:** Sin-Aye Park, Mee-Hyun Lee, Hye-Kyung Na, Young-Joon Surh

**Affiliations:** ^1^ Research Institute of Pharmaceutical Sciences, Seoul National University, Seoul 08826, South Korea; ^2^ Department of Molecular Medicine and Biopharmaceutical Sciences, Graduate School of Convergence Science and Technology, Seoul National University, Seoul 08826, South Korea; ^3^ Cancer Research Institute, Seoul National University, Seoul 110-799, South Korea; ^4^ Department of Food and Nutrition, College of Human Ecology, Sungshin Women's University, Seoul 136-742, South Korea

**Keywords:** 4-hydroxyestradiol, heme oxygenase-1, catechol estrogen, Nrf2, breast cancer

## Abstract

Estrogen (17β-estradiol, E_2_) undergoes oxidative metabolism by CYP1B1 to form 4-hydroxyestradiol (4-OHE_2_), a putative carcinogenic metabolite of estrogen. Our previous study showed that 4-OHE_2_-induced production of reactive oxygen species contributed to neoplastic transformation of human breast epithelial (MCF-10A) cells. In this study, 4-OHE_2_, but not E_2_, increased the expression of heme oxygenase-1 (HO-1), a sensor and regulator of oxidative stress, in MCF-10A cells. Silencing the *HO-1* gene in MCF-10A cells suppressed 4-OHE_2_-induced cell proliferation and transformation. In addition, subcutaneous administration of 4-OHE_2_ markedly enhanced the growth of the MDA-MB-231 human breast cancer xenografts, which was retarded by zinc protoporphyrin, a pharmacological inhibitor of HO-1. 4-OHE_2_-induced HO-1 expression was mediated by NF-E2-related factor 2 (Nrf2). We speculate that an electrophilic quinone formed as a consequence of oxidation of 4-OHE_2_ binds directly to Kelch-like ECH-associated protein 1 (Keap1), an inhibitory protein that sequesters Nrf2 in the cytoplasm. This will diminish association between Nrf2 and Keap1. 4-OHE_2_ failed to interrupt the interaction between Keap1 and Nrf2 and to induce HO-1 expression in Keap1-C273S or C288S mutant cells. Lano-LC-ESI-MS/MS analysis in MCF-10A-Keap1-WT cells which were treated with 4-OHE_2_ revealed that the peptide fragment containing Cys288 gained a molecular mass of 287.15 Da, equivalent to the addition of a single molecule of 4-OHE_2_-derived *ortho*-quinones.

## INTRODUCTION

4-Hydroxyestradiol (4-OHE_2_), an oxidized metabolite of 17β-estradiol (E_2_), is detected at considerably high levels in human breast tumors [1, 2]. This catechol estrogen has been proposed to contribute to hormonal carcinogenesis [3, 4]. 4-OHE_2_ with a catechol structure readily undergoes oxidation to electrophilic estradiol-3,4-quinone that can react with DNA to form depurinating adducts. Thus, estradiol-3,4-quinone may be an endogenous tumor initiator of mammary carcinogenesis [5–7]. The higher urinary levels of depurinating adducts derived from the 4-hydroxylated metabolite of estradiol were observed in women with breast cancer than in control subjects [8]. However, genotoxicity is not enough to complete the carcinogenic process, and the mechanisms underlying 4-OHE_2_-induced mammary carcinogenesis still remains poorly understood. In our previous study, reactive oxygen species (ROS) generated through redox cycling of 4-OHE_2_ induced the transformation of human breast epithelial (MCF-10A) cells [9]. Notably, ROS-mediated activation of the IκB kinase-NF-κB axis was found to be responsible for the transformation of these cells [9].

Heme oxygenase (HO) catalyzes degradation of heme to carbon monoxide, biliverdin, and free iron. Among three HO isoforms (HO-1, HO-2, and HO-3), HO-1 is the only inducible enzyme that plays a critical role in the cytoprotection against oxidative stress, inflammation, and other noxious stimuli [10–12]. However, the abnormally elevated expression and activity of this enzyme have been often reported in several tumor tissues, which may influence cancer cell proliferation, invasion and metastasis. In line with this notion, the overexpression of HO-1 enhanced viability, proliferation, and metastatic potential of melanoma cells [13]. HO-1 also stimulates angiogenesis through upregulation of vascular endothelial growth factor and overproduction of pro-angiogenic carbon monoxide [14]. Overexpression of HO-1 is detectable in biopsy tissues from oral AIDS-Kaposi sarcoma lesions [15] and advanced human prostate cancer tissues [16]. Moreover, pharmacological inhibitors of HO-1 have anti-carcinogenic effects in several tumor models [17, 18].

The transcription factor NF-E2-related factor 2 (Nrf2) regulates expression of genes encoding carcinogen detoxifying or antioxidative enzymes including HO-1 [19]. Nrf2 is present as an inactive complex in the cytoplasm with Kelch-like ECH-associated protein 1 (Keap1). One of the most plausible mechanisms responsible for Nrf2 activation involves modification of specific Keap1 cysteine residues [20]. This facilitates the liberation of Nrf2 from Keap1 and subsequently nuclear translocation, leading to induction of the antioxidant/electrophile responsive element (ARE/EpRE)-dependent target gene transcription. It is speculated that electrophilic quinones can activate Nrf2 by directly interacting with cysteine thiols of Keap1 [21, 22]. Sumi and colleagues have reported that quinoid metabolites derived from catechol estrogens covalently modify multiple Keap1 thiols as assessed by matrix-assisted laser desorption ionization time-of-flight mass spectrometry (MALDI-TOF MS) [23].

In the present study, we attempted to unravel the role of 4-OHE_2_-induced HO-1 overexpression in breast carcinogenesis and its underlying molecular mechanisms. We suggest that an electrophilic quinoid product derived from 4-OHE_2_ metabolism activates Nrf2 through Keap1 thiol modification and subsequently induces HO-1 upregulation, thereby stimulating proliferation, migration and malignant transformation of human mammary epithelial cells.

## RESULTS

### 4-OHE_2_ induces both expression and activity of HO-1

**Figure 1:**
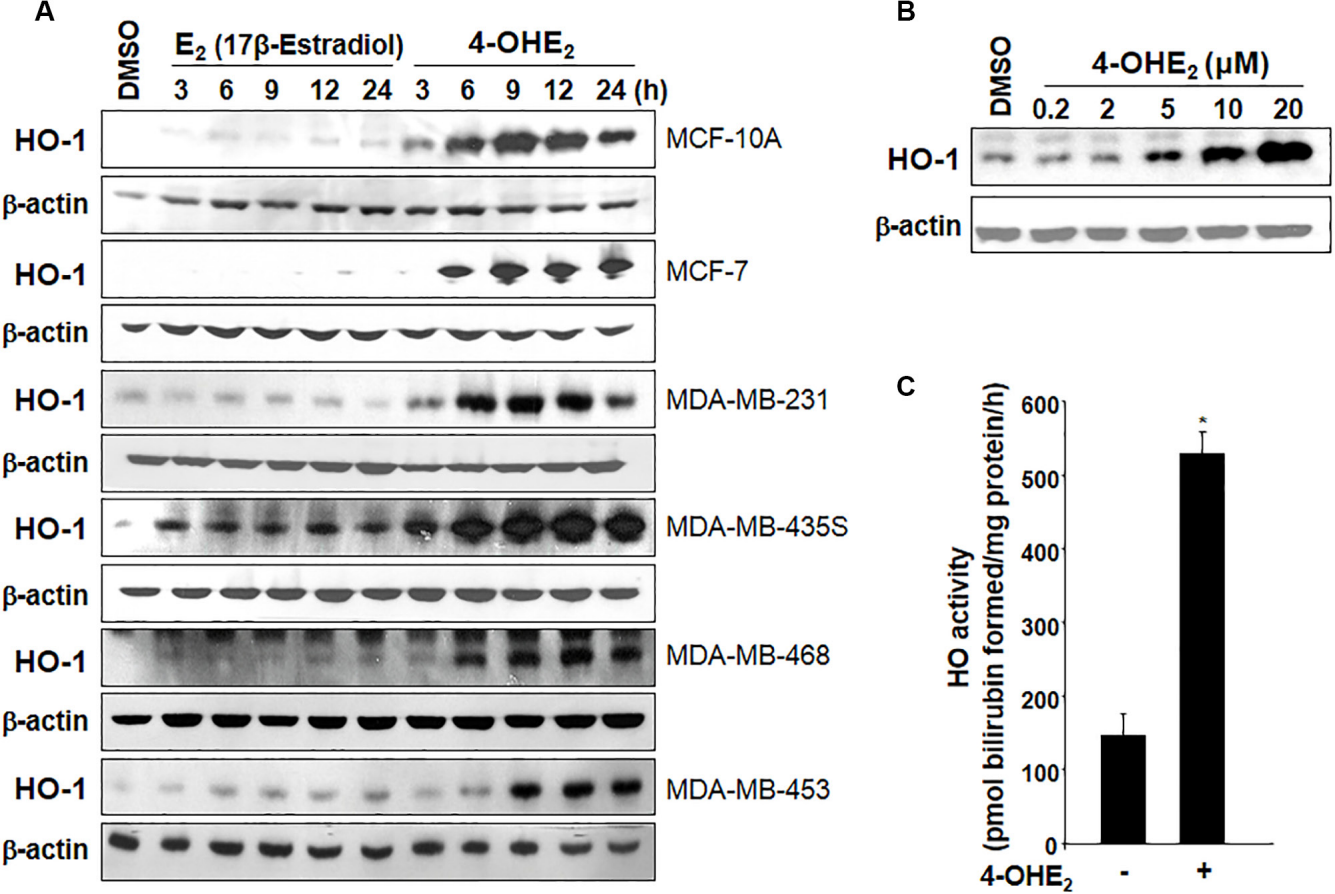
4-OHE_2_ induces both expression and/or activity of HO-1 in human breast cells (**A**) Each cell line was treated with E_2_ or 4-OHE_2_ (20 μM) for the indicated time periods, and the expression of HO-1 was assessed by Western blot analysis. (**B**) MCF-10A cells were treated with various concentrations of 4-OHE_2_ (0.2, 2, 5, 10, and 20 μM) for 6 h and the whole-cell lysates were subjected to Western blot analysis. (**C**) MCF-10A cells were treated with 4-OHE_2_ (20 μM) for 12 h and the HO activity was measured as described in Materials and Methods. *n* = 3; *P < 0.001.

The expression of HO-1 was significantly elevated by 4-OHE_2_, but not by the parent estrogen E_2_ in normal human breast epithelial MCF- 10A cells and several breast cancer cells (Figure 1A). The induction of HO-1 expression was concentration-dependent (Figure 1B). 4-OHE_2_ also increased the HO activity as measured by the amounts of bilirubin formed (Figure 1C).

### Inhibition of HO-1 expression or activity impairs 4-OHE_2_-induced cell proliferation

**Figure 2:**
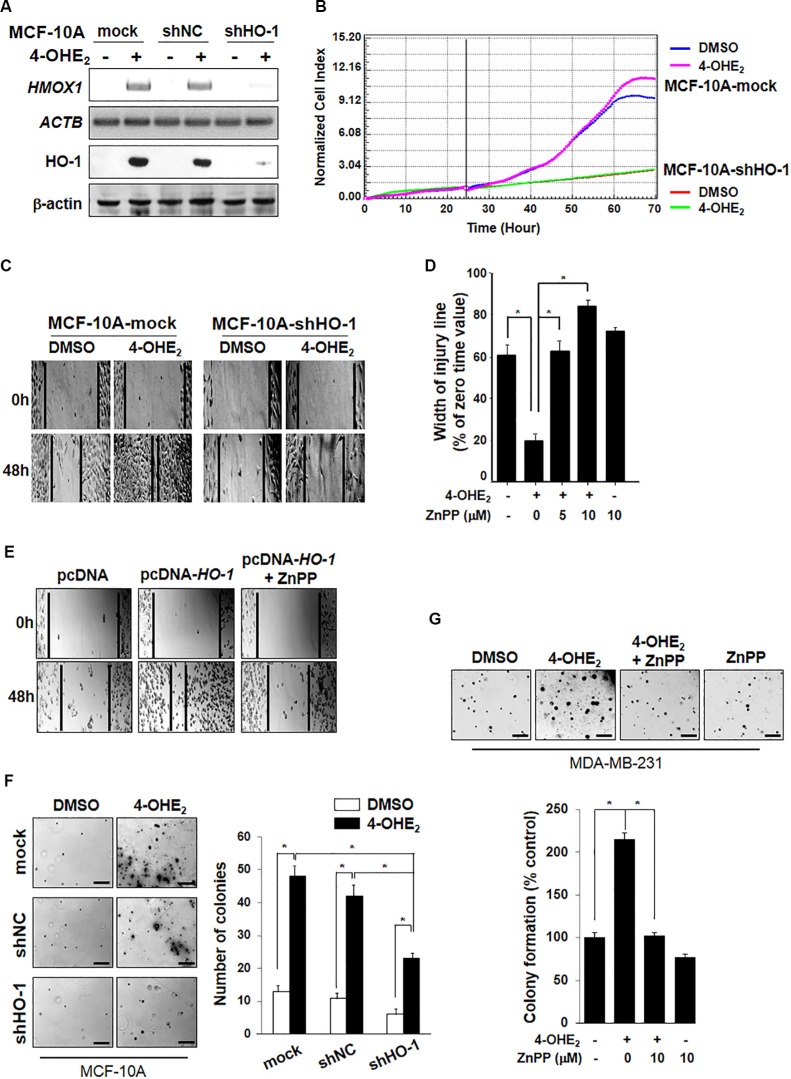
4-OHE_2_-induced HO-1 expression is associated with cell proliferation (**A**) MCF-10A cells stably expressing mock, negative control (NC) shRNA or HO-1 shRNA were selected with 0.5 μg/ml puromycin. Cells were exposed to 4-OHE_2_ (20 μM) for 6 h and analyzed for expression of HO-1 mRNA and protein by RT-PCR and Western blot analysis, respectively. *HMOX1*: HO-1 mRNA; *ACTB*: β-actin mRNA. (**B**) MCF-10A-mock or MCF-10A-shHO-1 cells were treated with DMSO or 4-OHE_2_ (5 μM) for 72 h, and the cell proliferation was measured as described in Materials and Methods. (C-E), Cell migration was measured by using Culture-Inserts, and the wound closure was monitored by photography at indicated time points as described in Materials and Methods. (**C**) The representative images of migration assay are from MCF-10A-mock or MCF-10A-shHO-1 cells treated with DMSO or 4-OHE_2_ (20 μM) for 48 h. (**D**) MCF-10A cells were treated with DMSO, 4-OHE_2_ (20 μM), or ZnPP (5 and 10 μM), separately or in combination for 24 h. *n* = 3; *P < 0.001. (**E**) The representative images of migration assay are from MDA-MB-231 cells transfected with control vector (pcDNA) or HO-1 plasmid in the absence or presence of ZnPP (10 μM) for 12 h. F-G, The anchorage-independent cell transformation assay was performed in MCF-10A or MDA-MB-231 cells as described in Material and Methods. Colonies were counted by using an inverted microscope (Nikon Diaphot 300). (**F**) MCF-10A-mock, MCF-10A-shNC, or MCF-10A-shHO-1 cells were treated with DMSO or 4-OHE_2_ (20 μM) once every 3 days for 3 weeks. Scale bars: 200 μm. *n* = 4; *P < 0.001. (**G**) MDA-MB-231 cells were treated with DMSO, 4-OHE_2_ (20 μM), or ZnPP (10 μM), separately or in combination. Scale bars: 200 μm. *n* = 4; *P < 0.001.

Previously, we reported that 4-OHE_2_ induced neoplastic transformation of MCF-10A cells [9]. To investigate the putative role of HO-1 in 4-OHE_2_-induced mammary epithelial cell transformation, MCF-10A cells stably expressing empty retroviral silencing vector (mock), negative control vector (shNC), or HO-1 shRNA (shHO-1) were utilized. As shown in Figure 2A, silencing of *HO-1* gene resulted in pronounced suppression of HO-1 protein expression in MCF-10A-shHO-1 cells. Notably, cells expressing HO-1 shRNA (MCF-10A-shHO-1) showed a significantly decreased proliferation after 24-h incubation in the absence or presence of 4-OHE_2_ (Figure 2B). In another experiment, the stable knockdown of HO-1 gene (*HMOX1*) in MCF-10A-shHO-1 cells retarded cell migration, compared with that in MCF-10A-mock cells in the absence or presence of 4-OHE_2_ (Figure 2C). Moreover, pharmacologic inhibition of HO-1 with zinc protoporphyrin IX (ZnPP) resulted in suppression of 4-OHE_2_-induced cell migration (Figure 2D). These results were further confirmed in a human breast cancer cell line (MDA-MB-231). When MDA-MB-231 cells were transfected with HO-1 plasmid (pc-DNA-*HO-1*), the cell migration was markedly enhanced which was reversed by ZnPP treatment (Figure 2E).

We next explored whether blockade of HO-1 could impair the transforming capability of 4-OHE_2_. Treatment of MCF-10A-mock or MCF-10A-shNC cells with 4-OHE_2_, twice a week for 3 weeks induced anchorage-independent cell growth, as evidenced by an increase in both the number and the size of colonies (Figure 2F). However, stable knockdown of HO-1 in MCF-10A-shHO-1 cells reduced colony formation more than 50% in the presence of 4-OHE_2_. Likewise, pharmacologic inhibition of HO-1 significantly reduced the 4-OHE_2_-induced anchorage-independent growth of MDA-MB-231 cells (Figure 2G). Taken together, these results suggest that HO-1 expression and activity contribute to the oncogenic potential of 4-OHE_2_ in human mammary epithelial cells.

### 4-OHE_2_ stimulates nuclear accumulation and ARE binding activity of Nrf2

**Figure 3:**
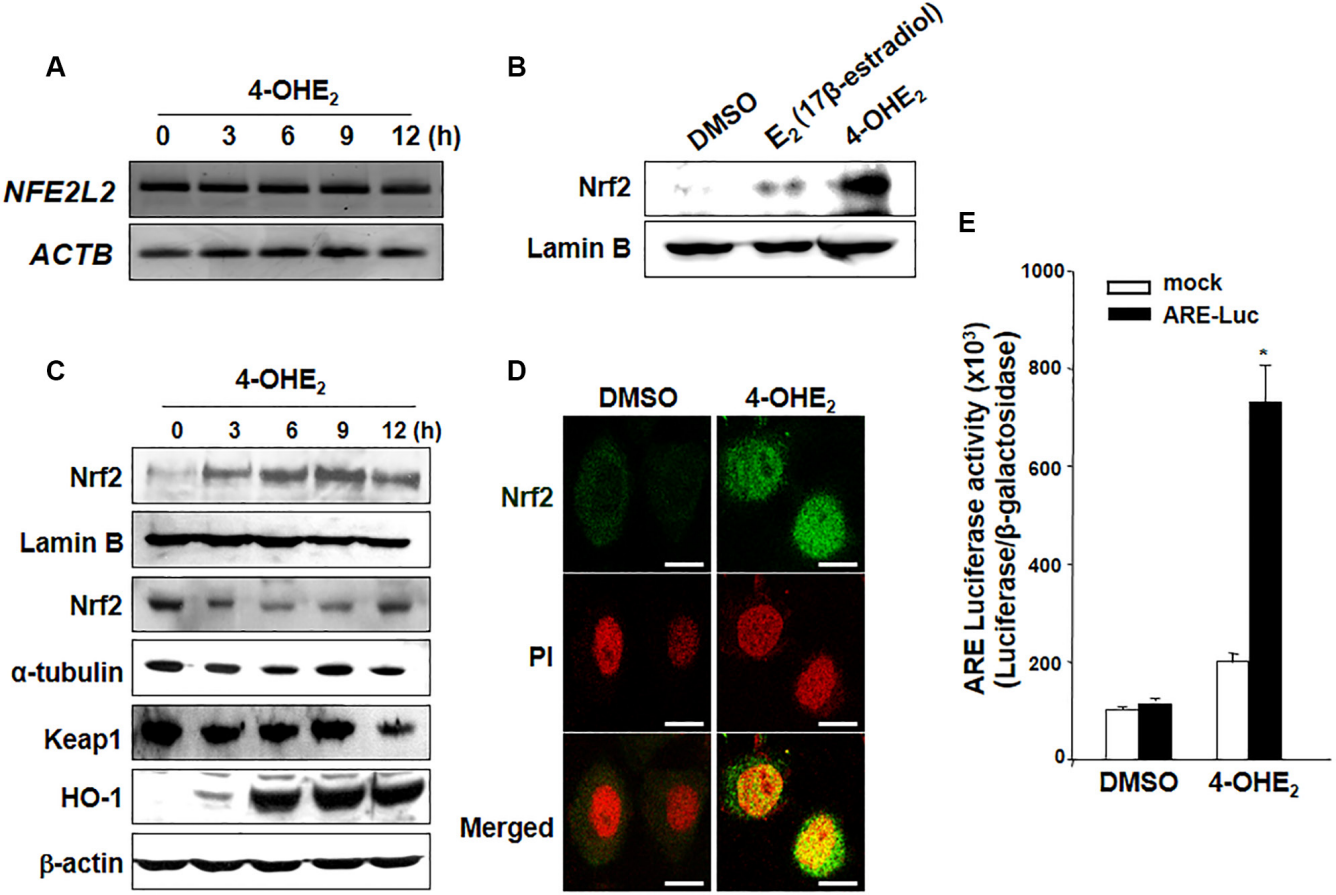
4-OHE_2_ induces the nuclear translocation of Nrf2 and transcriptional activity of ARE (**A**) MCF-10A cells treated with or without 4-OHE_2_ (20 μM) were harvested at the indicated intervals, and total RNA was analyzed by RT-PCR to check the level of Nrf2 mRNA. *NFE2L2*: Nrf2 mRNA; *ACTB*: β-actin mRNA. (**B**) MCF-10A cells were treated with E_2_ or 4-OHE_2_ (20 μM, each) for 3 h. The nuclear extracts were separated and subjected to Western blot analysis. (**C**) Nuclear, cytosol or whole extracts from MCF-10A cells were prepared at the indicated intervals after treatment with 4-OHE_2_ (20 μM), followed by Western blot analysis. (**D**) After the cells were treated with DMSO or 4-OHE_2_ (20 μM) for 3 h, immunofluorescence staining of Nrf2 was conducted as described in Materials and Methods. Scale bars: 10 μm. (**E**) MCF-10A cells were co-transfected with pCMV-β-galactosidase and either the luciferase reporter gene fusion construct (pTi-luciferase) or WT ARE and for 18 h, followed by treatment with DMSO or 4-OHE_2_ for 6 h. The cells were analyzed for the ARE transcriptional activity as described in Materials and Methods. *n* = 3; *P < 0.001.

It was previously reported that 4-OHE_2_ activated Nrf2 via the phosphatidylinositol-4,5-bisphosphate 3-kinase (PI3K) pathway [24]. As HO-1 induction is primarily regulated by Nrf2-ARE signaling, we first examined the effect of 4-OHE_2_ on the expression of Nrf2. 4-OHE_2_ failed to alter *Nrf2* mRNA (*NFE2L2*) expression (Figure 3A) but it enhanced nuclear translocation of Nrf2 more strongly than did the parent compound E_2_ (Figure 3B). 4-OHE_2_-induced nuclear accumulation of Nrf2 was accompanied by transient reduction in its cytoplasmic levels followed by the increased expression of HO-1 (Figure 3C). 4-OHE_2_-induced accumulation of Nrf2 in the nucleus of MCF-10A cells was also verified by immunocytochemical analysis (Figure 3D). Additionally, we examined the effect of 4-OHE_2_ on Nrf2 transcriptional activity by use of the ARE luciferase reporter gene assay. The induction of ARE luciferase activity (> 7-fold) was observed after 6-h treatment of 4-OHE_2_ (Figure 3E).

### 4-OHE_2_-induced HO-1 expression is dependent on Nrf2 activation

**Figure 4:**
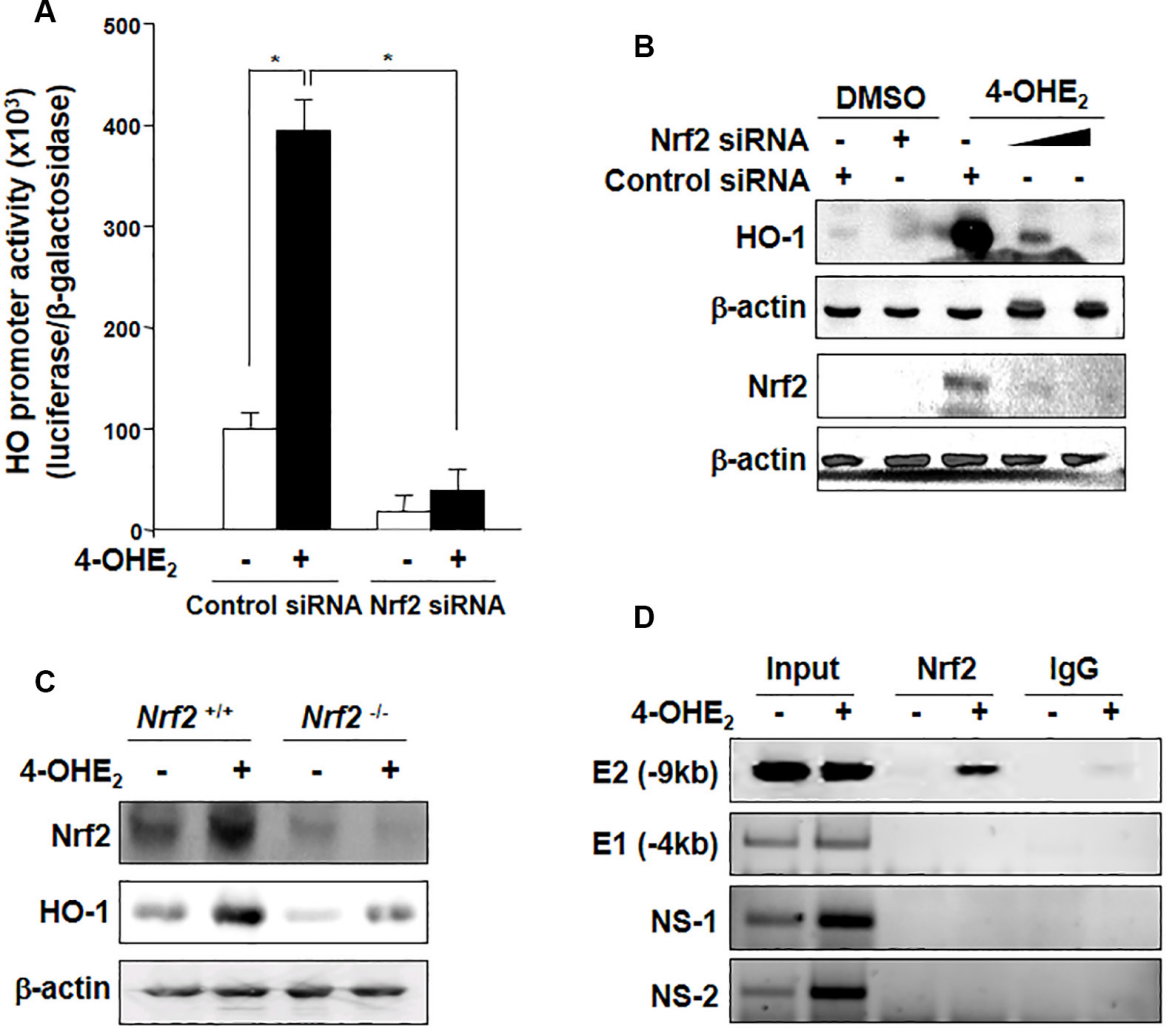
4-OHE_2_-induced HO-1 expression is mediated through Nrf2 activation (**A**) MCF-10A cells were co-transfected with luciferase reporter plasmid construct harboring the HO-1 binding site (pGL2-HO-1) and pCMV-β-galactosidase with control siRNA or Nrf2 siRNA (20 nM) for 18 h, followed by treatment with 4-OHE_2_ for additional 6 h. 4-OHE_2_-mediated transcriptional activation of HO-1 was measured by the luciferase reporter assay as described in Materials and Methods. *n* = 3; *P < 0.001. (**B**) Cells were transfected with control siRNA or Nrf2 siRNA (20 nM) for 18 h and exposed to 4-OHE_2_ (20 μM) for another 6 h. The cell lysates were subjected to Western blot analysis. (**C**) The effect of Nrf2 on the expression of HO-1 was assessed by using embryonic fibroblasts from *Nrf2* WT (*Nrf2*^+/+^) and *Nrf2*-null (*Nrf2*^−/−^) mice. The cells were exposed to 4-OHE_2_ (20 μM) for 6 h and subjected to Western blot analysis. (**D**) MCF-10A cells were treated with DMSO or 4-OHE_2_ (20 μM) for 6 h and harvested for the ChIP assay. Chromatin immunoprecipitated DNA was analyzed by PCR with primers for distal E2 (−9.0 kb region) ARE, E1 (−4.0 kb region) ARE, and two non-specific regions (NS-1 and NS-2) of the *HO-1* promoter.

To determine whether 4-OHE_2_-induced HO-1 up- regulation is mediated by Nrf2, we examined the level of HO-1 expression after Nrf2 knockdown. The 4-OHE_2_-induced promoter activity (Figure 4A) and expression (Figure 4B) of HO-1 were abolished by silencing Nrf2 gene with its specific siRNA. To further verify the role of Nrf2 in HO-1 induction, we utilized primary embryonic fibroblasts obtained from the Nrf2 wild-type (WT; *Nrf2*^+/+^) and null (*Nrf2*^−/−^) mice. As shown in Figure 4C, 4-OHE_2_-induced HO-1 expression was blunted in the fibroblasts derived from *Nrf2^−/−^* mice. We also employed the chromatin immunoprecipitation (ChIP) assay to more precisely assess Nrf2 interaction with the HO-1 gene promoter in the presence of 4-OHE_2_. There was the strong binding of Nrf2 to only the distal E2 (−9.0 kb) region of the HO-1 promoter harboring the ARE consensus sequence upon exposure to 4-OHE_2_ (Figure 4D). Taken together, these results support that 4-OHE_2_-induced HO-1 expression is regulated mainly by Nrf2.

### Cysteine 288 of Keap1 is a putative target of 4-OHE_2_ for HO-1 induction

**Figure 5:**
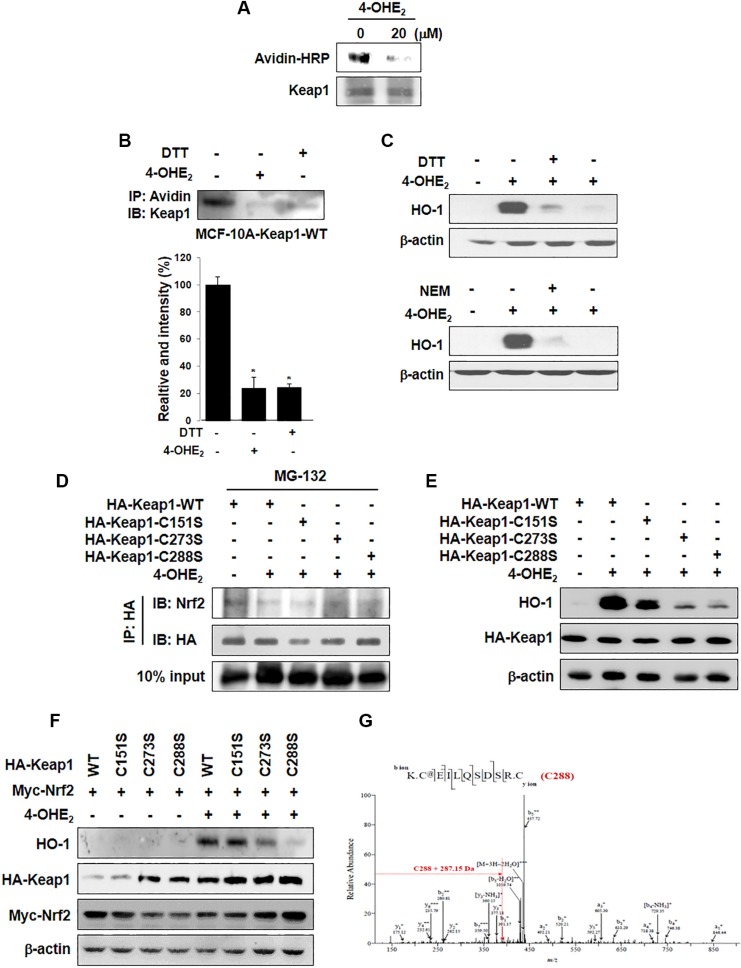
The thiol modification of Keap1 is responsible for 4-OHE_2_-induced Nrf2 activation and HO-1 expression (**A**) Recombinant human Keap1 protein was reacted with DMSO or 4-OHE_2_ (20 μM) for 30 min, and then incubated with BPM (50 μM) for additional 30 min. The resulting protein was subjected to Western blot analysis with an anti-biotin antibody. (**B**) MCF-10A-Keap1 WT cells were incubated with DMSO, 4-OHE_2_ (20 μM), or DTT (100 μM) for 1 h and then lysed with RIPA buffer. The cell lysates were subjected to the BPM-labeling assay as described in Materials and Methods. *n* = 3; *P < 0.001. (**C**) MCF-10A cells were pre-treated with DTT (100 μM) or NEM (25 μM) for 1 h, followed by 6 h incubation with 20 μM 4-OHE_2_. Whole cell lysates were subjected to Western blot analysis. (**D**) MCF-10A cells stably expressing HA-Keap1-WT, HA-Keap1-C151S, HA-Keap1-C273S, or HA-Keap1-C288S were pre-treated with MG-132 (10 μM) for 1 h and then treated with DMSO or 4-OHE_2_ (20 μM). The proteins were lysed with RIPA buffer and immunoprecipitated with anti-HA antibody. The immunoprecipitated proteins were subjected to Western blot analysis with an anti-Nrf2 or anti-HA antibody. (**E**) MCF-10A-Keap1 WT or each mutant cells were incubated with DMSO or 4-OHE_2_ (20 μM) for 6 h, and the whole cell lysates were assessed by Western blot analysis. (**F**) MCF-10A cells were co-transfected with an expression vector for Myc-Nrf2 and an expression vector for either WT Keap1 or mutant Keap1 for 18 h. The transfected cells were treated with DMSO or 4-OHE_2_ (20 μM) for additional 6 h, and then cell lysates were subjected to Western blot analysis. (**G**) MCF-10A cells stably expressing WT Keap1 were incubated with DMSO or 4-OHE_2_ (20 μM) for 1 h, and the proteins were subjected to in-gel digestion. Digested peptide fragments were subjected to mass spectrometry as described in Materials and Methods.

Upon its cysteine thiol modification, Keap1 liberates Nrf2 that translocates into the nucleus. We speculate that an electrophilic quinone species formed as a consequence of oxidation of 4-OHE_2_ may directly bind and subsequently modify cysteine thiol(s) of Keap1, thereby facilitating the dissociation of Nrf2. The previous MALDI-TOF MS analysis has revealed that multiple reactive thiol groups of recombinant mouse Keap1 protein are subjected to modification by quinoid metabolites derived from catechol estrogens [23]. Here, we found that incubation of recombinant human Keap1 with 4-OHE_2_ reduced the level of Keap1 modified by biotin-PEAC_5_-maleimide (BPM), indicative of 4-OHE_2_ binding to Keap1 (Figure 5A). 4-OHE_2_ caused a decline in the band intensity for BPM bound to Keap1 in MCF-10A cells, corroborating the covalent modification of Keap1 by this catechol estrogen (Figure 5B). Dithiothreitol (DTT), a well-known thiol reducing agent, also abrogated the interaction between BPM and Keap1. In another experiment, treatment of MCF-10A cells with DTT or a disulfide alkylating agent *N*-ethylmaleimide (NEM) repressed 4-OHE_2_-induced HO-1 expression in MCF-10A cells (Figure 5C).

Of the cysteine residues present in Keap1, Cys151, Cys273, and Cys288 are known to function as redox sensors [25–27]. To identify which cysteine residue of Keap1 is a primary target of 4-OHE_2_, we constructed HA-tagged retroviral mutant vectors in which each of above cysteine residues was replaced by serine (C151S, C273S, and C288S). The stable cell lines expressing each of these mutant constructs as well as the WT cell line were treated with MG132, a cell-permeable proteasome inhibitor, 1 h before 4-OHE_2_ treatment. Cell lysates were immunoprecipated with the antibody directed against the HA-Keap1, and the immunoprecipitated proteins were subjected to immunoblot analysis. The interaction between Keap1 and Nrf2 was not interrupted by 4-OHE_2_ in Keap1-C273S or -C288S mutant cells (Figure 5D), and 4-OHE_2_ failed to induce HO-1 expression in these cells (Figure 5E). Moreover, induction of HO-1 expression by 4-OHE_2_ was not prominent in cells which were co-transfected with expression vectors for Myc-Nrf2 and Keap1-Cys273S or Keap1-Cys288S (Figure 5F). To identify the specific cysteine residue(s) modified by 4-OHE_2_, MCF-10A cells overexpressing WT Keap1 were treated with this catechol estrogen. The proteins were digested with trypsin, and the resulting peptides were analyzed by nano-LC-ESI-MS/MS. An increment of 287.15 Da corresponding to the addition of a single molecule of 4-OHE_2_-derived *ortho*-quinone was observed in the peptide fragment containing Cys288 (Figure 5G). These results suggest that cysteine 288 of Keap1 is likely to be a potential target of 4-OHE_2_ for its activation of Nrf2-HO-1 signaling.

### Inhibition of HO-1 activity attenuates the growth of 4-OHE_2_-enhanced human breast cancer cell xenograft

**Figure 6:**
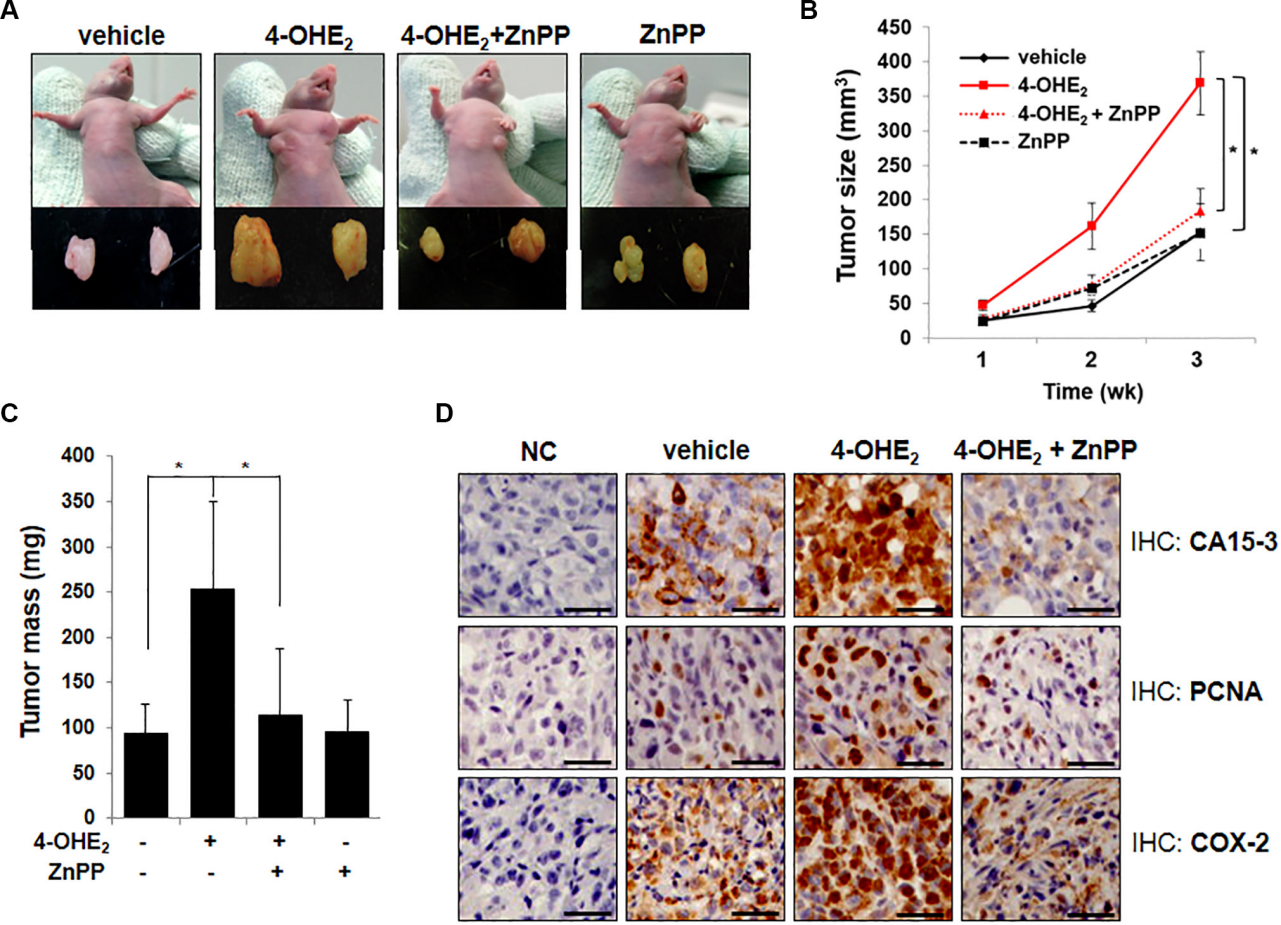
Inhibition of HO-1 activity by ZnPP impairs 4-OHE_2_-induced tumorigenesis in mice (**A**) The representative images of human mammary tumor (MDA-MB-231) xenografts in mice treated with vehicle, 4-OHE_2_, 4-OHE_2_ plus ZnPP, or ZnPP for 3 weeks (*n* = 12/group). (**B**) The effect of each treatment on the tumor volume of MDA-MB-231 cell xenograft was measured with digital calipers and calculated by the formula 0.52 × length × width^2^. Vehicle vs 4-OHE_2_ or 4-OHE_2_ vs 4-OHE_2_ + ZnPP; *P < 0.001 (Two-sided *t*-test). (**C**) The effects of each treatment on the tumor mass were measured. *P < 0.001 (Two-sided *t*-test). (**D**) The effect of each treatment on the expression of the indicated genes was examined by immunohistochemical analysis as described in Materials and Methods (Magnification, × 400). NC: negative control, Scale bars: 50 μm.

Because HO-1 appears to mediate the carcinogenic activity of 4-OHE_2_, we examined whether suppression of HO-1 activity could affect the 4-OHE_2_-induced growth of MDA-MB-231 human mammary cancer cells transplanted to athymic mice. As shown in Figure 6A, tumor growth was increased in mice receiving 4-OHE_2_, but significantly suppressed by co-treatment with ZnPP. Thus, the average volume (Figure 6B) and mass (Figure 6C) of tumors in 4-OHE_2_-treated mice were almost completely reduced by pharmacologic inhibition of HO-1. It is noticeable that the enhanced growth of MDA-MB-231 cell xenografts by 4-OHE_2_ treatment was associated with a marked increase in the expression levels of CA15-3 (Figure 6D), which is a typical diagnostic marker of breast tumor. Pharmacologic inhibition of HO-1 with ZnPP also reduced the CA15-3 expression and suppressed the tumor cell proliferation as evidenced by decreased expression of proliferating cell nuclear antigen (PCNA). Inappropriate up-regulation of cyclooxygenase-2 (COX-2) correlates with the increased angiogenesis and metastatic potential in breast cancer cells [28, 29]. 4-OHE_2_-induced COX-2 overexpression in MDA-MB-231 human breast tumor xenografts was also inhibited by ZnPP treatment (Figure 6D). Taken together, these data suggest that HO-1 plays a critical role in 4-OHE_2_-mediated breast cancer progression.

## DISCUSSION

It has been reported that HO-1 is overexpressed in several human tumors [30–32]. In this study, we found that 4-OHE_2,_ one of the putative oncogenic metabolites of E_2_, upregulated the expression of HO-1 in human breast epithelial cells and multiple breast cancer cells. HO-1 expression was induced by 4-OHE_2_, not by the parent compound E_2_, through activation of Nrf2 which might result from the direct binding of 4-OHE_2_-derived electrophilic quinone species to Cys288 of Keap1. This 4-OHE_2_-mediated thiol modification of Keap1 activates Nrf2-HO-1 signaling, which contributes to enhanced proliferation and transformation of mammary epithelial cells, and stimulation of breast cancer cell growth.

The oncogene-induced Nrf2 transcription has been proposed to promote tumorigenesis [33], and the elevated expression/activity of HO-1, one of the major Nrf2 target proteins, can affect tumor cell proliferation, invasion, metastasis, and chemoresistance [13, 15, 34, 35]. Our previous study has demonstrated that 15-deoxy-Δ^12,14^-prostaglandin J_2_, formed during COX-2-catalyzed arachidonic acid metabolism, upregulates HO-1 expression and subsequently provokes ROS formation, which may account for increased invasiveness and metastatic potential of human breast cancer cells [36]. HO-1 knockdown led to pronounced inhibition of the pancreatic cancer cell growth and made tumor cells significantly more sensitive to radiotherapy and chemotherapy [31]. Cobalt protoporphyrin (CoPP), an inducer of HO-1 expression and activity, enhanced the vGPCR-induced tumor growth while a HO-1 inhibitor SnPP treatment caused a remarkable reduction [37]. Moreover, ZnPP and its water-soluble pegylated form (PEG-ZnPP) have been found to act as anti-neoplastic agents when given to mice bearing tumors [17, 38].

Some cysteine residues present in Keap1 that functions as redox sensors are recognized as potential targets of thiol-reactive chemical species capable of inducing Nrf2 activation [20, 25, 26, 39]. It has been reported that mutation of Cys151, Cys273, or Cys288 negates Keap1 functional activity [25, 26, 39–42]. Covalent modification of Cys273 or Cys288 induces conformational changes in the intervening region (IVR) of Keap1 and affects the interaction between IVR of Keap1 and the Neh2 domain of Nrf2 while modification of Cys151 does not impair recognition of Nrf2, but rather causes dysfunction of the E3 ligase [27]. Notably, our mass spectral analysis indicates that 4-OHE_2_ directly interacts with the Cys288 residue of an endogenous form of Keap1 expressed in human breast epithelial cells, suggesting that this particular cysteine could be a *bona fide* target of this catechol estrogen in its activation of Nrf2 and upregulation of HO-1 expression.

Several studies have reported that mitogen-activated protein kinases (MAPKs) and Akt are involved in Nrf2 activation [43–45]. 4-OHE_2_ was shown to activate the ARE by a PI3K/Akt-dependent mechanism [24]. ROS overproduced by 4-OHE_2_ was found to cause the increased phosphorylation of extracellular signal-regulated kinase (ERK) and Akt [9]. Results from our preliminary study demonstrate the ROS-mediated activation of PI3K- Akt signaling as an alternative mechanism underlying 4-OHE_2_-induced Nrf2 activation and HO-1 upregulation (Supplementary Figure 1).

In conclusion, our data show that 4-OHE_2_ induces upregulation of HO-1 through Nrf2 activation, and genetic or chemical inhibition of this enzyme abrogates 4-OHE_2_-induced mammary epithelial cell transformation and growth of tumor xenografts. These results provide a novel molecular mechanism of 4-OHE_2_-induced progression of mammary carcinogenesis. Considering that HO-1 is overexpressed in several cancers including breast cancer, the targeted inhibition of intratumoral HO-1 activity and/or expression may be a potential clinical interest in future anticancer therapy.

## MATERIALS AND METHODS

### Reagents

4-OHE_2_ and E_2_ were purchased from Sigma Chemical Co. Rabbit polyclonal HO-1 antibody was purchased from Stressgen. The primary antibody of Nrf2, Keap1, and PCNA were purchased from Santa Cruz Biotechnology. Anti-CA 15–3 was obtained from Fitzgerald. The primary antibody for COX-2 was purchased from Neomarker. ZnPP was supplied from Alexis Corporation. Matrigel basement membrane matrix was purchased from BD Bioscience. HO-1 short interfering RNA (siRNA) was purchased from Invitrogen. HO-1 plasmid was kindly provided by Prof. Jozef Dulak (Jagiellonian University, Krakow, Poland). *N*-Acetyl-L-cysteine (NAC), trolox, DTT, NEM and an antibody against actin were also purchased from Sigma Chemical Co. The primary antibodies of lamin B and α-tubulin and anti-rabbit horseradish peroxidase-conjugated secondary antibody were products of Zymed Laboratories Inc. The inhibitors of ERK (U0126) and PI3K (LY294002) were purchased from TOCRIS. BPM was obtained from DOJINDO. Human recombinant Keap1 protein was purchased from Abnova. Avidin agarose was a product of Pierce. The ECL chemiluminescent detection kit was obtained from Amersham Pharmacia Biotech. The human specific *Nrf2* siRNA (sense 5′-AAGAGUAUGAGCUGGAAAAACTT-3′; antisense 5′-GUUUUUCCAGCUCAUAC UCUUTT-3′) and Stealth^TM^ RNAi negative control duplexes were provided by Invitrogen. The luciferase assay kit was purchased from Promega.

### Cell culture

MCF-10A, MCF-7, MDA-MB-231, MDA-MB-435S, MDA-MB-453, and MDA-MB-468 cells were originally obtained from American Type Culture Collection and cultured according to the manufacturer's instruction. All cells were passaged directly from the original low-passage stocks and were used before passage 30. The cells were examined within the last three months for correct morphology under a microscope and tested to detect mycoplasma contamination using an e-Myco^TM^ plus mycoplasma polymerase chain reaction (PCR) detection kit (iNtRON Biotechnology). Cells are maintained at 37°C in a humidified atmosphere composed of 5% CO_2_/95% air.

### Reverse transcription PCR (RT-PCR)

Total RNA was isolated from cells using TRIzol^®^ (Invitrogen). One microgram of total RNA was used for the complementary DNA synthesis using random primers. RT-PCR was performed following standard procedures. PCR conditions for *NFE2L2* (Nrf2) and *ACTB* (β-actin) were as follows: 26 cycles of 94°C for 1 min; 60°C for 1 min and 72°C for 1 min. The primer pairs and the size of the expected products were as follows: *NFE2L2*, 5′-CGGTATGCAACAGGACATTG-3′ and 5′-ACTGGTTGGGGTCTTCTGTG-3′, 263 bp and *ACTB*, 5′-CTCTTCCAGCCTTCCTTCCT-3′ and 5′- AGC ACTGTGTTGGCGTACAG-3′, 211 base pairs. Amplification products were resolved by 1.0% agarose gel electrophoresis, stained with ethidium bromide and photographed under ultraviolet light.

### Western blot analysis

Standard SDS-PAGE and Western blotting procedures were used to analyze the expression of various proteins. Cell lysates were prepared using SDS lysis buffer (50 mM Tris-HCl, pH 6.8, 2% SDS, 10% glycerol, and 0.02% bromophenol blue) containing protease inhibitors and phosphatase. All proteins were visualized using a horseradish peroxidase-conjugated secondary antibody and Amersham ECL™ Western Blotting Detection Reagents (GE Healthcare Life Sciences).

### ChIP assay

DNA and proteins of the cells were cross-linked with 37% formaldehyde for 10 min at room temperature. The cells were washed with ice-cold phosphate-buffered saline (PBS) containing protease inhibitor cocktail tablets (Roche Molecular Biochemicals), scraped in PBS, and centrifuged at 2,000 *g* for 5 min. Pellets were suspended in SDS lysis buffer [1% SDS, 10 mM EDTA, 50 mM Tris (pH 8.1)] with protease inhibitor cocktail tablets. Lysates were sonicated to 200- to 1000-bp in length on ice and centrifuged at 13,000 × *g* for 10 min. ChIP dilution buffer containing protease inhibitor cocktail tablets was mixed with the supernatant chromatin. Each sample was immunoprecipitated with 5 μg of specific Nrf2 antibody or normal mouse IgG overnight at 4°C rotation. Immune complexes were precipitated with protein G agarose beads (Santa Cruz Biotechnology) for 4 h at 4°C with rotation. The pellets were washed once with low salt immune complex wash buffer ([0.1% SDS, 1% Triton X-100, 2 mM EDTA, and 20 mM Tris-HCl (pH 8.1), and 150 mM NaCl], once with high salt immune complex wash buffer [0.1% SDS, 1% Triton X-100, 2 mM EDTA, 20 mM Tris-HCl (pH 8.1), and 500 mM NaCl], once with LiCl immune complex wash buffer [0.25 M LiCl, 0.5% NP-40, 1% deoxycholic acid, 1 mM EDTA, and 10 mM Tris-HCl (pH 8.1)], and twice with TE buffer [10 mM Tris-HCl (pH 8.1), and 1 mM EDTA]. DNA-protein complexes were eluted from protein G agarose beads with an elution buffer [0.1 M NaHCO_3_ and 1% SDS]. Cross-linking was reversed at 65°C overnight, and DNA was extracted using the *AccuPrep* Genomic DNA Extraction Kit (Bioneer) according to the manufacturer's protocol. PCR was performed against the E2 ARE of the HO-1 promoter (primers; 5′-CCCTGCTGAGTAATCCTTTCCCGA-3′ and 5′-ATGTCCCGACTCCAGACTCCA-3′), and the PCR condition was as follows: 40 cycles of 94°C for 1 min, 60°C for 1 min, and 72°C for 1 min. PCR was also performed against the E1 ARE (primers; 5′-CTGCCCAAACC ACTTCTGTT-3′ and 5′-ATAAGAAGGCCTCGGTGG AT-3′) and two non-specific (NS) ARE regions of the HO-1 promoter (NS-1 primers; 5′-GCTATGTGGGAGGTT GAGGA-3′ and 5′-CCATGGTCAGCAGTTTGCTA-3′, NS-2 primers; 5′-TTGCCTTGTCACGTTTTCAC-3′ and 5′-TGCCTTGGTGTCTCAGAGTG-3′). The PCR condition was as follows: 40 cycles of 94°C for 1 min, 50°C for 1 min, and 72°C for 1 min.

### Site-directed mutagenesis

Site-directed mutagenesis was performed by PCR using DNA primers obtained from Bioneer with single-, double- or triple-based mismatches, resulting in the desired amino acid substitution according to the manufacturer's instruction (Intron Biotechnology). The correct sequence of all constructs was confirmed by sequencing analysis (Cosmo Genetech).

### Measurement of HO-1 activity

HO activity was measured by determining the rate of bilirubin production according to the previously described method [46, 47]. Harvested cells were washed twice with cold PBS, scraped in the presence of cold homogenization buffer containing 30 mM Tris-HCl (pH 7.5), 0.25 M sucrose, 0.15 M NaCl, and a mixture of protease inhibitors, homogenized, and centrifuged at 10,000 × *g* for 15 min at 4°C. The supernatant was collected and centrifuged again at 100,000 × *g* for 1 h at 4°C. The obtained microsomal pellet was resuspended in 100 mM potassium phosphate buffer (pH 7.4) containing 2 mM MgCl_2_, 10 μg/ml leupeptin, 10 μg/ml trypsin inhibitor, 2 μg/ml aprotinin, and 1 mM phenylmethylsulfonyl fluoride. This microsomal fraction was added to the reaction mixture containing 0.8 mM NADPH, 2 mM glucose 6-phosphate, 0.2 U of glucose-6-phosphate dehydrogenase, 20 μM hemin, 100 mM potassium phosphate buffer (pH 7.4), and 2 mg of rat liver cytosol as a source of biliverdin reductase. Mixtures were incubated at 37°C for 1 h in the dark, and the samples were left in an ice bath for at least 2 min to terminate the reaction. Bilirubin formed was determined by calculation of the differences in absorbance between 464 and 530 nm.

### Generation of cells expressing HO-1 shRNA

The sequences of HO-1 shRNA and negative control shRNA (shNC) obtained from OriGene Technologies were as follows: HO-1 shRNA, TCCTTAC ACTCAGCTTTCTGGTGGCGACA and shNC, TGACCA CCCTGACCTACGGCGTGCAGTGC. Each sequence was cloned into a retroviral silencing (pRS) vector. Cells stably expressing original pRS vector (mock), shNC or HO-1 shRNA were selected with 0.5 μg/ml puromycin (Invivogen).

### Cell proliferation assay

MCF-10A-mock or MCF-10A-shHO-1 cells were plated at a density of 2 × 10^3^ cells/well in the E-Plate 96 (Roche Diagnostics Corporation) for 24 h. Each of the 96 wells on the E-Plate 96 contains integral sensor electrode arrays so that cells inside each well can be monitored. The cells were incubated for another 72 h in the absence or presence of 4-OHE_2_ in the Real-Time Cell Analyzer (Roche Diagnostics Corporation).

### Wound migration assay

The Culture-Inserts (Ibidi) were transferred to 6-well plates, and cells (7 × 10^4^/well) were seeded in Culture-Inserts. After 24-h incubation, the Inserts were removed and cells were treated with the medium containing each agent. Phase contrast images of the closed gap were captured at the indicated time of incubation using an inverted microscope (magnification, ×10).

### Anchorage-independent growth assay

To prepare hard agar layer, 3.3% agarose dissolved in PBS was boiled, and the agarose solution was added to 60 mm-dishes and kept in the 37°C incubator to solidify. To prepare the soft agar layer, cells were suspended in the 0.33% agarose solution, and the mixture was inoculated on top of the hard agar layer. After 4-h incubation, fresh media was added to the top of the soft agar layer, and the cells were exposed to 4-OHE_2_ or vehicle, once in 3 days. After incubation for 3 to 4 weeks, anchorage-independent growth (spherical formation containing >10 cells) was scored under a light microscope. The experiments were replicated 4 times, and a representative set of data was photographed.

### Luciferase reporter assay

The cells were seeded in a 6-well plates and grown to 60–70% confluence. MCF-10A cells were co-transfected with pCMV-β-galactosidase and either the luciferase reporter gene fusion construct (pTi-luciferase) or WT ARE. Cells were also co-transfected with a luciferase reporter plasmid construct harboring the HO-1 binding site (pGL2-HO-1) and pCMV-β-galactosidase with control siRNA or Nrf2 siRNA. After 18-h transfection, the medium was changed, and cells were treated with 4-OHE_2_ for additional 6 h. The cells were then washed with PBS and lysed in reporter lysis buffer (Promega). The lysed cell extract was mixed with the luciferase assay reagent, and the luciferase activity was determined using a luminometer (AntoLumat LB 953). The β-galactosidase assay (Promega) was done to normalize the luciferase activity.

### Preparation and maintenance of mouse embryonic fibroblasts

The Nrf2 wild type (*Nrf2*^+/+^) and Nrf2-null (*Nrf2*^−/−^) mice were provided by Dr. Jeffery Johnson (University of Wisconsin, Madison, USA). After in-house breeding, the *Nrf2*^−/−^, *Nrf2*^+/−^ and WT mice were maintained in the animal quarters in accordance with Seoul National University guidelines for animal care and were housed in a 12-h light/12-h dark cycle. Male and female *Nrf2*^+/−^ mice were mated and embryos were obtained at the day 13.5 after pairing under aseptic conditions. The heads of the embryos were used to confirm the *Nrf2* genotype by RT-PCR, and the embryo bodies were minced into small pieces and cultured in high glucose DMEM supplemented with 10% FBS and kept at 37°C with 5% CO_2_.

### Immunofluorescence staining

The cells were rinsed with PBS and fixed with 4% formaldehyde for 30 min at room temperature. After washing with PBS, the fixed cells were incubated in PBS containing 10% BSA and 0.5% Tween-20 for 2 h at room temperature. After incubation with an Nrf2 antibody (1:100 dilution) overnight at 4°C, the cells were washed with PBS and labeled with diluted (1:1000) FITC-conjugated goat anti-rabbit IgG (Zymed Laboratories) for additional 1 h at room temperature. After washing with PBS, cells were stained with propidium iodide (PI), visualized under a confocal microscope and photographed (Leica Microsystems Heidelberg GmbH).

### Generation of stable cells expressing Keap1 constructs

pBabe parental vector (pBabe-puro-HA-VHL) was purchased from Addgene and Keap1-C151S, -C273S, and −C288S cDNAs were obtained using Muta-Direct^TM^ (iNtRON Biotechnology). PCR-amplified WT Keap1, Keap1-C151S, −C273S, or −C288S cDNA was subcloned into the parental vectors. Recombinant retroviruses were produced as described previously [48]. Briefly, HEK293T cells were transiently transfected with each retroviral construct and packaging vectors (gag-pol and VSVG). After 24-h transfection, the media were changed, and the virus containing media was collected at 48 h and filtered with 0.45 μm syringe filter (Whatman). Target cells were incubated with the retrovirus-containing media with 8 μg/ml polybrene (Millipore) and selected with puromycin (1 μg/ml).

### Detection of Keap1 modified by 4-OHE_2_

The BPM-labeling assay was conducted to determine the modified Keap1 thiol groups after exposure to 4-OHE_2_ based on the protocol described by Abiko *et al.* [49]. In the cell-free system, recombinant human Keap1 protein was reacted with 4-OHE_2_ (20 μM) for 30 min at 25°C in 20 mM Tris-HCl (pH 8.5), followed by incubation with 50 μM BPM for 30 min at 37°C and the resulting protein was subjected to Western blot analysis. In another experiment, the same assay was conducted with MCF-10A cells expressing Keap1 WT. In brief, cells were washed with PBS and lysed with RIPA buffer (50 mM Tris-HCl, pH 8; 0.1% SDS; 150 mM NaCl; 1% NP- 40; and 0.5% deoxycholic acid). The cell lysates were centrifuged, and the supernatant was incubated with 100 μM BPM for 30 min at 37°C. The resulting lysates were immunoprecipitated by using avidin-agarose overnight at 4°C. After being washed, the precipitated proteins were eluted and analyzed by Western blot analysis with anti-Keap1 antibody.

### Enzymatic in-gel digestion

Briefly, protein samples isolated from DMSO or 4-OHE_2_-treated MCF-10A-Keap1 WT cells were loaded on 4–12% gradient Novex Bis-Tris gel and the gel was stained with GelCode^®^ Blue Stain Reagent (Thermo scientific). For the alkylation of the separated proteins, 55 mM iodoacetamide was added and incubated for 30 min in the dark at room temperature. The alkylated protein samples were digested using trypsin (Promega) overnight at 37°C and the digested peptides were extracted with 5% formic acid in 50% acetonitrile solution. The supernatants were collected and dried with Speed Vac (Thermo Scientific). The samples resuspended in 0.1% formic acid were purified and concentrated using C18 ZipTips (Millipore) before mass spectrometry (MS).

### Analysis by nano-liquid chromatography-electrospray ionization-tandem mass spectrometry (LC-ESI-MS/MS)

The tryptic peptides were analyzed using an LTQ (Thermo Finnigan) ion trap equipped with a nano-ESI source in the positive ion mode. Peptides were injected onto a homemade C18 packed reversed phase column (12 cm × 75 μm) and were eluted with a linear gradient from 3 to 50% solvent B (0.1% formic acid in acetonitrile) at a flow rate of 200 nL/min. The electrospray voltage and the threshold for switching MS to MS/MS were set to 1.95 kV and 500, respectively. The normalized collision energy was 35% of the main RF amplitude for MS/MS. All spectra were acquired in a data-dependent scan. Five most intense peaks from the full MS scan were fragmentized. The following parameters were used: repeat count for dynamic exclusion = 1, repeat duration = 30 s, dynamic exclusion duration = 180 s, exclusion mass = 1.5 Da, and the size of the dynamic exclusion list = 50.

### Tumor growth in a xenograft model

Female BALB/c (*nu*/*nu*) athymic nude mice, 6 weeks of age, were purchased from Charles River Laboratories. All animal works were approved by the Seoul National University Ethics Research Board. MDA-MB-231 cells (3 × 10^6^ in 100 μl PBS plus 100 μl matrigel) were injected subcutaneously into the mammary fatpad regions. After 5 days of implantation, mice were separated in groups of six animals and were subcutaneously treated with vehicle, ZnPP (10 μmol/kg of body weight dissolved in 0.1 N NaOH in PBS, pH 7.5), 4-OHE_2_ (0.3 μg/0.1 ml/mouse) alone or together with ZnPP, daily for 3 weeks. Tumor volume (0.52 × length × width^2^) and body weight were measured three times a week.

### Immunohistochemical analysis

Slides using 4 μm sections of formalin-fixed and paraffin-embedded xenograft tumors were prepared for immunohistochemical analysis as described previously [50]. Slides were incubated separately with primary antibodies for CA 15-3 (1:200), PCNA (1:500), or COX-2 (1:500) and developed using the anti-rabbit or anti-mouse horseradish peroxidase Envision System (DAKO). The counterstaining was done using Mayer's hematoxylin.

### Data analysis

The acquired LC-ESI-MS/MS fragment spectra were searched in the BioWorksBrowser^TM^ (version Rev. 3.3.1 SP1, Thermo Scientific) with the SEQUEST search engines against the data in FASTA format generated from Keap1 (NCBI accession number NM_203500.1). The conditions for the search were as follows; trypsin as enzyme specificity, a permissible level for two missed cleavages, peptide tolerance; ±2 amu, a mass error of ±1 amu on fragment ions and variable modifications of carbamidomethylation of cysteine (+57 Da), oxidation of methionine (+16 Da) and 4-OHE_2_-induced modification of cysteine (+287.15 Da) residues.

### Statistical evaluation

Data were expressed as the mean ± SD of the results obtained from at least three independent experiments. Statistical significance of the obtained data was determined by conducting Student's *t*-test, and a *p*-value of less than 0.01 was considered to be statistically significant.

## SUPPLEMENTARY MATERIALS


