# Correction: 4-Hydroxyestradiol induces mammary epithelial cell transformation through Nrf2-mediated heme oxygenase-1 overexpression

**DOI:** 10.18632/oncotarget.26681

**Published:** 2019-02-08

**Authors:** Sin-Aye Park, Mee-Hyun Lee, Hye-Kyung Na, Young-Joon Surh

**Affiliations:** ^1^ Research Institute of Pharmaceutical Sciences, Seoul National University, Seoul 08826, South Korea; ^2^ Department of Molecular Medicine and Biopharmaceutical Sciences, Graduate School of Convergence Science and Technology, Seoul National University, Seoul 08826, South Korea; ^3^ Cancer Research Institute, Seoul National University, Seoul 110-799, South Korea; ^4^ Department of Food and Nutrition, College of Human Ecology, Sungshin Women's University, Seoul 136-742, South Korea

**This article has been corrected:** During the assembly of Figure 2F, the same image was inadvertently used for both the mock and nonspecific RNA control (shNC) in vehicle (DMSO) treated groups. The proper Figure 2 is shown below. The authors declare that these corrections do not change the results or conclusions of this paper.

**Figure 2:**
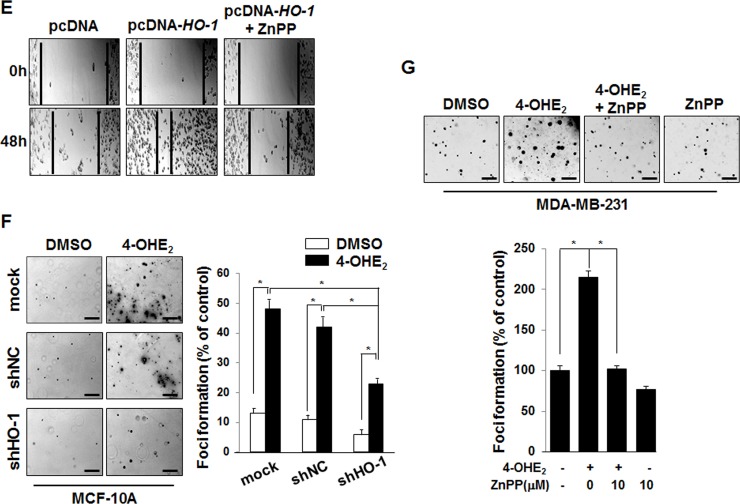
4-OHE_2_-induced HO-1 expression is associated with cell proliferation (**E**) The representative images of migration assay are from MDA-MB-231 cells transfected with control vector (pcDNA) or HO-1 plasmid in the absence or presence of ZnPP (10 μM) for 12 h. F-G, The anchorage-independent cell transformation assay was performed in MCF-10A or MDA-MB-231 cells as described in Material and Methods. Colonies were counted by using an inverted microscope (Nikon Diaphot 300). (**F**) MCF-10A-mock, MCF-10A-shNC, or MCF-10A-shHO-1 cells were treated with DMSO or 4-OHE2 (20 μM) once every 3 days for 3 weeks. Scale bars: 200 μm. *n* = 4; *P < 0.001. (**G**) MDA-MB-231 cells were treated with DMSO, 4-OHE2 (20 μM), or ZnPP (10 μM), separately or in combination. Scale bars: 200 μm. *n* = 4; *P < 0.001.

Original article: Oncotarget. 2017; 8:164-178. https://doi.org/10.18632/oncotarget.10516

