# Correction: Cancer therapeutic approach based on conformational stabilization of mutant p53 protein by small peptides

**DOI:** 10.18632/oncotarget.26921

**Published:** 2019-05-07

**Authors:** Perry Tal, Shay Eizenberger, Elad Cohen, Naomi Goldfinger, Shmuel Pietrokovski, Moshe Oren, Varda Rotter

**Affiliations:** ^1^ Department of Molecular Cell Biology, The Weizmann Institute of Science, Rehovot, Israel; ^2^ Department of Molecular Genetics, The Weizmann Institute of Science, Rehovot, Israel

**This article has been corrected:** Due to errors during figure assembly, several mistakes occurred in the legends and presentation of Figures 1, 5 and 6. The authors declare that these corrections do not change the results or conclusions of this paper.

They are as follows:

1. In Figure 1C, one of the lanes has been inadvertently duplicated.

2. In Figure 5H, a dark square was inadvertently introduced in one of the images.

3. In Figure 5L, there was a mix-up in some of the panels. Since the conclusions from this panel reiterate the conclusions from panels G and H immediately above panel L, panel L has been removed.

4. The description of panels D and E was added to the legend of Figure 6.

The corrected figures and legends are shown below:

**Figure 1:**
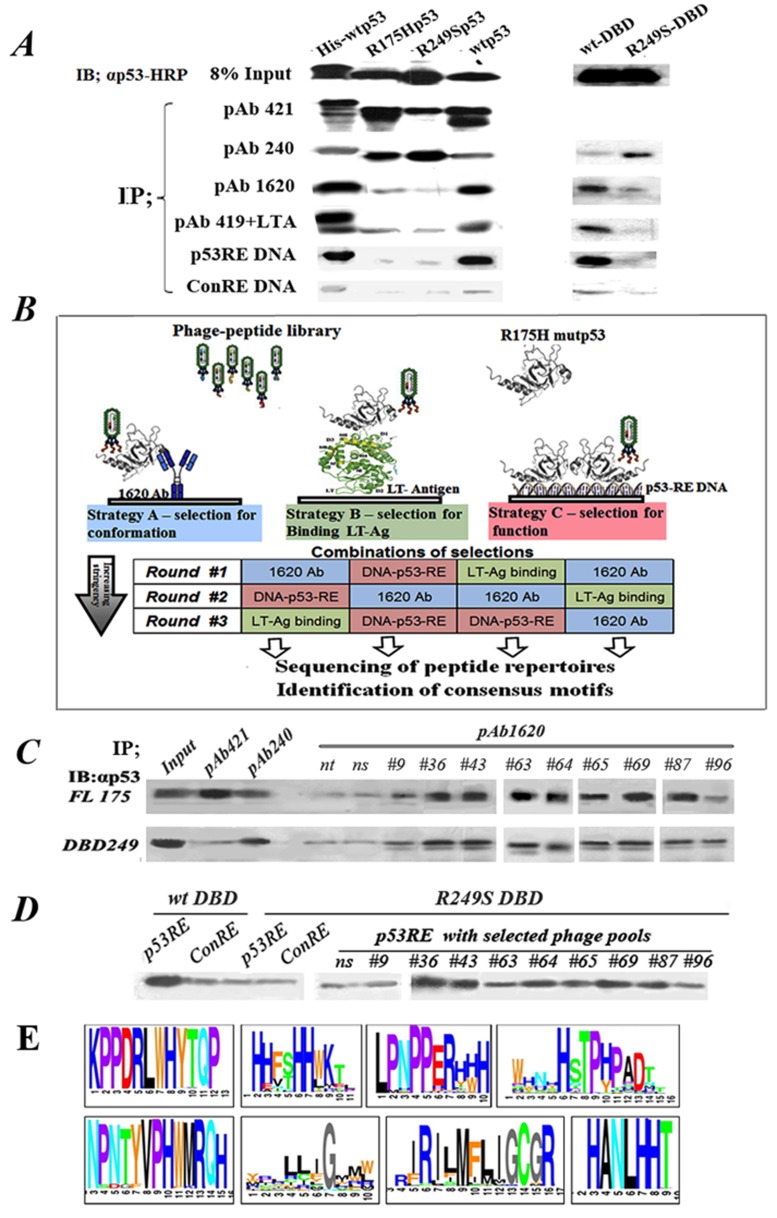
Outline of experimental rationale, calibration of conditions. **A.** IP-Western analysis of the binding of WTp53 and mutp53 to various markers distinguishing between WT and mutp53 conformations. 50ng of each purified protein was subjected for immunoprecipitation with a subset of different antibodies and proteins shown to bind differentially WTp53 or mutp53: PAb421 (binds both WT and mutant), PAb1620 (WT specific), PAb240 (mutant specific), PAb419+LTag (WT specific), biotinilated-p53RE DNA oligo (WT specific), and a control oligo mutated at two bases. Immunoprecipitated material was subjected to Western blotting using αp53-HRP as second antibody. **B.** Schematic diagram representing the protocol for identification, screening and selection of mutp53 reactivating peptides. The protocol consists of various selection strategies, at increasing stringencies, for screening and identifying mutp53 reactivating peptides, by utilizing phage display. Strategy A (left): Conformation-based selection: selection of peptides presented by a phage, which can bind a mutp53 protein bound to immobilized WTp53 conformation-specific antibody (PAb1620), thereby enabling selection of a bound phage capable of stabilizing WTp53 conformation. Strategy B (middle): selection according to binding: selection of peptides, which can bind a mutp53 protein bound to immobilized LT-antigen. Strategy C (right): selection according to function: selection of peptides binding to immobilized WTp53-p53RE DNA complex. **C.** Western blot analysis of IP with PAb1620 antibody of purified either p53^R175H^ (upper panel) or p53^R249S^-DBD (lower panel) in the presence of selected phage pools. Non-selected phage (ns) and no phage (nt) were used as controls. Incubation was for 3 hours at 4°C. Bound p53 in the immunoprecipitate (IP) was analyzed by Western blot using antibody against p53 (αp53). “In” stands for 10% of the IP input material, loaded directly on the gel. **D.** Western blot analysis of IP experiments of streptavidin- coated beads bound either to p53RE-DNA or control-RE-DNA oligonucleotides labeled with biotin were incubated with purified WTp53- DBD or mutant p53^R249S^-DBD in the presence of phage selected by phage display. Non selected phage (ns) were used as control. Incubation was for 3 hours at 4oC. **E.** A schematic illustration of several consensus peptide motifs identified as described.

**Figure 5:**
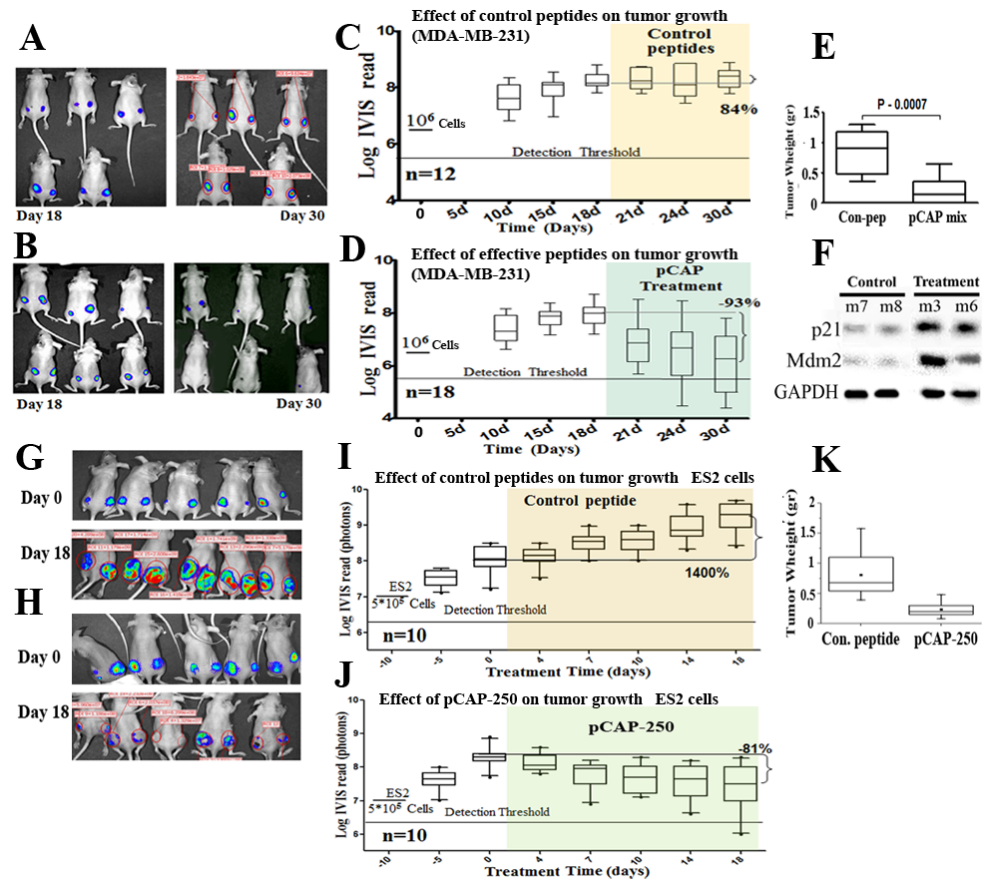
Effect of lead peptides on tumor growth in vivo; breast and ovarian cancer models. **A.–D.** - Effect of indicated peptides in a mouse breast cancer xenograft model.10^6^ MDA-MB-231 breast cancer cells, expressing endogenous mutp53 and stably expressing luciferase, were injected into the hips of nude mice. When tumors reached visible size, mice were treated by intra-tumoral injection, three times a week, with either a mixture of 3 control peptides that showed no phenotype *in vitro * (pCAPs 76, 77 and 12; 2μg of each peptide) or a mixture of 3 test peptides that exhibited mutp53-reactivating ability (pCAPs D60R, 24R and 174; 2μg of each peptide). **A. -** Live imaging of control group mice at the beginning of treatment (day 18) and at termination of the experiment (day 30). **B. -** Live imaging of mice treated with effective peptide mix at the beginning of treatment (day 18) and at termination of the experiment (day 30). **C. -** (control group mice) and **D.** (effective pCAP mix group): Logarithmic scale box-plot showing the luciferase readings in tumors as a function of time; average (horizontal line), standard deviation (box), highest and lowest reads (error bars) are shown, before (until day 18) and after initiation of treatment. The background threshold detection level of the IVIS system in this experiment was about 5x106 photons. **E. -** Box-plot of excised tumor weights at termination of the experiment. Average weight (horizontal line), standard deviation (box), highest and lowest reads are shown. **F.-** Western blot analysis of two p53 target gene products, p21 and MDM2. Part of the excised tumors of mice #7 and #8 treated with control peptides and mouse #3 treated with effective peptide mix were homogenized and lysed. Protein concentration was determined using Bio-Rad reagent. 50μg protein of each sample was loaded and subjected to Western blot analysis with antibodies against p21 and MDM2. **G.–K. -** In-vivo effect of indicated peptides in a mouse xenograft model. 5^*^10^5^ ES2 cells expressing luciferase were injected into the hips of nude mice. Bioluminescence was measured. 10 days after injection, mice were randomly divided to 2 groups and injected intratumorally, three times a week, with either a mixture of 2 control peptides (pCAPs 76 and 12; 5μg of each peptide) or pCAP- 250 (10μg). G., **H. -** Live imaging of control group mice and pCAP-250 treated mice, respectively, at the beginning of treatment (day 0) and at termination of experiment (day 18). **I.** (control mice) and **J.** (effective pCAP-250 group): box-plot showing the luciferase readings in tumors as a function of time; average (horizontal line), standard deviation (box), highest and lowest reads are shown, before (until day 0) and after initiation of treatment. The background threshold detection level of the IVIS system was about 5x106 photons. **K. -** Box-plot of excised tumor weights at termination of the experiment. Average weight (horizontal line), standard deviation (box), highest and lowest reads are shown.

**Figure 6:** Effect of lead peptides on tumor progression *in vivo *; colon cancer xenograft model. *In vivo * effect of the indicated peptides in a mouse xenograft model of SW-480 colon cancer cells. 106 cells were injected and tumors were allowed to establish for 10 days. Mice were then randomly divided into 3 groups and injected intratumorally, three times a week, with either a mixture of 3 control peptides (pCAPs 76, 77 and 12; 2μg of each peptide), a mixture of 3 test peptides that exhibited mutp53-reactivating ability (pCAPs 250, 308 and 325; 2μg of each peptide) or pCAP-325 (6μg) . **A.–C.** - Logarithmic scale graph demonstrating the average luciferase readings in tumors as a function of time, before and after initiation of treatment (colored background). **D., E.** - Live imaging of control group mice (D) and the group treated with peptide mix (E) at termination of the experiment (day 35). **G., H.**- Box plot of tumor volume and weight, respectively.

Original article: Oncotarget. 2017; 8:11817–11837. https://doi.org/10.18632/oncotarget.10516

