# Supplementary material for: 4-Hydroxyestradiol induces mammary epithelial cell transformation through Nrf2-mediated heme oxygenase-1 overexpression
Source: Oncotarget. 2016 Jul 9;8(1):164–78. doi: 10.18632/oncotarget.10516 (PMC5352084; doi:10.18632/oncotarget.10516)
Supplement: Supplementary file 1 [file oncotarget-08-164-s001.pdf]

4-Hydroxyestradiol induces mammary epithelial cell transformation through Nrf2-mediated heme oxygenase-1 overexpression

Supplementary Materials

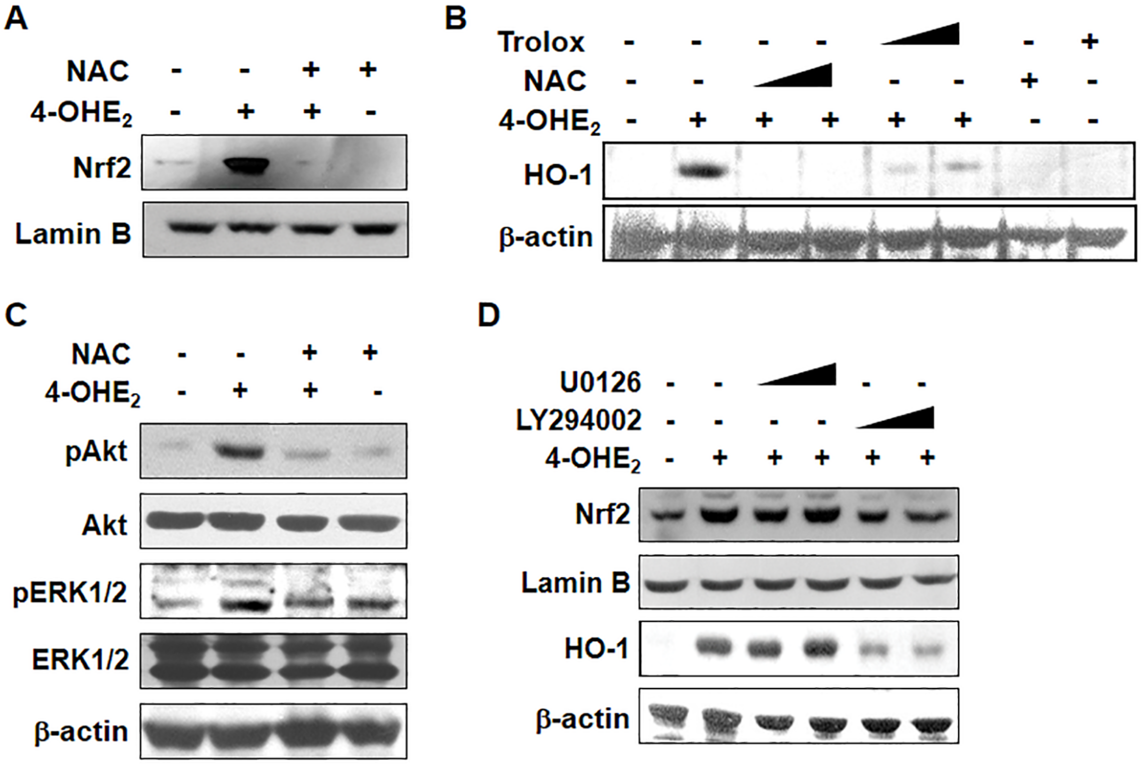

Supplementary Figure S1: ROS-mediated Akt phosphorylation is involved in 4-OHE$_2$-induced Nrf2 activation and HO-1 expression. (**A**) The effect of NAC on nuclear translocation of Nrf2 in 4-OHE$_2$-treated MCF-10A cells. MCF-10A cells were exposed to 20 µM 4-OHE$_2$ for 3 h in the absence or presence of NAC (5 mM), and subjected to Western blot analysis. (**B**) The effect of NAC and trolox on the expression of HO-1 in 4-OHE$_2$-treated cells. MCF-10A cells were exposed to 20 µM 4-OHE$_2$ for 6 h in the absence or presence of NAC (3 or 5 mM) or trolox (25 or 50 µM). (**C**) MCF-10A cells incubated with 20 µM 4-OHE$_2$ for 3 h in the absence or presence of NAC (5 mM), and cell lysates were assessed by Western blot analysis to detect phosphorylated and total Akt and ERK. (**D**) Cells were pretreated with U0126 (12.5 or 25 µM), a pharmacological inhibitor of ERK or the PI3K/Akt inhibitor LY294002 (12.5 or 25 µM) for 1 h, followed by exposure to 4-OHE$_2$ for 3 h or 6 h to measure Nrf2 or HO-1, respectively.
